# Evaluation of the Staff Educational Components of the PROMOTE Program to Improve Resident Hydration

**DOI:** 10.3390/nu16223861

**Published:** 2024-11-12

**Authors:** Heather H. Keller, Raksha Aravind, Kristina Devlin, Safura Syed, Sophia Werden Abrams, Christina Lengyel, Minn N. Yoon, Ashwini Namasivayam-MacDonald, Susan E. Slaughter, Phyllis Gaspar, Wen Liu

**Affiliations:** 1 Kinesiology and Health Sciences, University of Waterloo, 200 University Ave W, Waterloo, ON N2L 3G1, Canada; r4aravind@uwaterloo.ca (R.A.); kristina.devlin@uwaterloo.ca (K.D.); safura.syed@uwaterloo.ca (S.S.); 2 School of Rehabilitation Science, McMaster University, 1280 Main St W, Hamilton, ON HSC 3H9, Canada; werdenan@mcmaster.ca (S.W.A.); namasia@mcmaster.ca (A.N.-M.); 3 Department of Food and Human Nutritional Sciences, University of Manitoba, 35 Chancellors Circle, Winnipeg, MB R3T 2N2, Canada; christina.lengyel@umanitoba.ca; 4 Mike Petryk School of Dentistry, University of Alberta, 5-575 Edmonton Clinic Health Academy, 11405-87 Avenue NW, Edmonton, AB T6G 1C9, Canada; minn.yoon@ualberta.ca; 5 Faculty of Nursing, University of Alberta, 11405-87 Ave, Edmonton, AB T6G 1C9, Canada; susan.slaughter@ualberta.ca; 6 Independent Consultant, 27020 Rolling Thunder Lane, Sioux Falls, SD 57108, USA; pgaspar1976@gmail.com; 7 College of Nursing, The University of Iowa, 50 Newton Road, Iowa City, IA 52317, USA; wen-liu-1@uiowa.edu

**Keywords:** long-term care, retirement home, hydration, education, staff, fluid consumption

## Abstract

Background/Objectives: Inadequate fluid intake is prevalent among older adults living in care settings and can lead to dehydration-related events such as falls and hospitalization. Staff knowledge and confidence using diverse strategies is needed to provide adequate hydration to residents. PROMOTE is a multicomponent intervention designed to support staff to increase resident fluid intake between meals. This study evaluated the educational components of PROMOTE. Methods: Participants (*n* = 87) working in long-term care or retirement homes completed an online pre-/post-test evaluation of a 7 min educational video. Key informant participants (*n* = 13) reviewed all educational materials, evaluated their usefulness and feasibility, and were interviewed to identify how to improve the materials. Results: The educational video improved knowledge (e.g., self-rating of knowledge pre-test median 8, standard error of the mean (SEM) 0.18; post-test median 9, SEM 0.13) and confidence. Participants intended to use PROMOTE strategies in their work with residents (1 [very likely] to 10 [very unlikely] median 2.0 SEM 0.27). Key informants rated the hydration of residents as an organizational priority (median 9.0 SEM 0.42) and all indicated that they would use the educational video in their future training. Less feasible educational components as rated by key informants included huddle discussions and email pushes. Posters were seen as feasible (54%) but only somewhat useful (77%). Conclusions: Brief educational videos can improve staff knowledge and confidence regarding providing adequate hydration to residents. Having several educational components that can be used with this video was viewed positively. Recommendations were made to improve the materials.

## 1. Introduction

Older adults (i.e., residents) living in long-term care (LTC) and other seniors’ living settings (e.g., retirement homes) are at high risk for dehydration. Dehydration and impending dehydration are often under-recognized, but their prevalence is estimated at up to 50% of residents [1]. Hypernatremia, most often due to low fluid intake, is more common in patients admitted to hospital from LTC compared to community-living older adults [2]. Resident fluid intake is typically low—for example, 51% of residents were found to consume less than 8 oz of fluid at a single meal [3]. There are several reasons for low intake, including inadequate access to beverages between meals; a decreased thirst drive; fear of incontinence; requiring thickened fluids; cognitive impairment; lack of staff monitoring; infections; renal and cardiovascular diseases; or being at the end of life [2,3,4,5,6]. Fluid in the form of two or more beverage choices is typically provided at meals but may compete with food intake during meals. Offering fluid between meals provides the opportunity to increase fluid intake [7]. Multi-component interventions to improve fluid intake between meals are recommended to address the varying reasons for low fluid intake among LTC residents [8]. However, it is essential to evaluate each component individually to determine effectiveness.

PROMOTE, a multi-component intervention to increase fluid intake between meals, has been created based on evidence and acceptability of previously published strategies [9]. To develop PROMOTE strategies, diverse staff (*n* = 162) reviewed 20 strategies that tap into various reasons why older adults do not consume enough fluids in care settings and rated these strategies for acceptability and feasibility [9]. Based on these findings, a draft description of the PROMOTE intervention was created. Staff (*n* = 25) reviewed and provided feedback of these PROMOTE strategy descriptions in a qualitative interview, considering how these could be implemented in LTC [10]. Revisions to the PROMOTE intervention resulted from these consultations. PROMOTE stands for seven key strategies to increase fluid intake between meals, as follows: **P**referred drinks and cups/glasses, with the specific requirement that vessels be easy to hold and contain at least 250 mL of fluid; **R**eminders to drink, which includes direct care staff meetings or huddles to discuss how to promote fluid intake, with discussion guides provided for the team lead, as well as email pushes to remind staff of PROMOTE key messages; **O**ffer fluid with other care routines, events, and activities such as when a resident is having their teeth brushed or after their physiotherapy session; **M**edication fluid offers, where the nursing team provides fluid with pills and encourages residents to drink a full glass of fluid at that time; **O**ffer fluids as a social opportunity, recognizing that residents drink more when with others; **T**rolley of drink choices, where three or more beverages, including a choice of thickened beverages, are provided to residents between meals; and **E**ducate and encourage, which includes an educational video for staff, micro-videos on these strategies, and educational and motivational posters for staff and residents/family.

Staff education and training are essential to multi-component interventions like PROMOTE. Staff members are routinely involved in offering fluids and encouraging residents to eat and drink, and thus should be aware of strategies that can work. To support optimal resident hydration, staff also need to understand resident fluid needs, the importance of hydration, and signs and symptoms of dehydration [11]. Furthermore, behavioural interventions, such as encouraging or prompting residents to drink, have been shown to be effective in increasing resident hydration [12]. These behavioural interventions also require trained direct care staff. Prior research on hydration interventions, specifically in LTC, is limited, and few interventions have included or focused on evaluating the component that educates staff on hydration needs and strategies [12,13].

Prior research suggests that peer education can be effective at increasing fluid intake in individuals with incontinence [14], while others which used awareness-raising workshops improved staff knowledge but did not increase the fluid intake of residents [15]. Details on educational methods were generally poorly described [14,15] and evaluation specifically of the education components of these multi-component interventions was not completed [14,15]. Intensive interactive education in the form of a 2 h multi-activity program (I-Hydrate) improved knowledge, but not all staff were reached due to the time commitment [16]. As a result, these authors provided a 15 min huddle discussion on hydration strategies, but this was not formally evaluated [16]. E-learning appears to be a viable means of reaching staff and improving knowledge, allowing staff to complete modules at their convenience [17]. These studies suggest that staff education on hydration, alone and as part of a multi-component intervention, are important to improve hydration, but that further development and evaluation are needed. Specifically, brief asynchronous video education has not been evaluated.

During the COVID-19 pandemic, a multi-component intervention with strategies resulting from direct care staff input was developed and implemented by members of this research team. Education specifically had to be simple and easy to implement for many staff at once. The multi-component intervention, named “Wet Your Whistle with Water”, included a 6 min voluntary asynchronous video focused on how much residents should drink, why they do not drink enough, and strategies to support water intake [18]. Other components included poster reminders (displayed on screens in common areas), a hydration station, and fluid offerings with recreational events. The educational video was evaluated with pre- and post-test questions, and knowledge based on four questions showed improvement [18]. However, confidence and intention to change behaviours post education were not evaluated, nor were the poster reminders evaluated. Although other multi-component interventions [15,19] describe awareness campaigns, such as those used in “Wet Your Whistle With Water”, it is not clear what was communicated in these campaigns and how well they were received.

PROMOTE is an evolution of the “Wet Your Whistle With Water” program [18] and includes several educational components to be used in combination to educate staff on using the seven PROMOTE strategies. These educational components include one 7 min animated video for staff members; fourteen physical paper posters; eight email push reminders; eight 2 min animated micro-videos based on a single hydration topic (e.g., medication reminders to drink); and eight huddle-topic discussions focused on the key messages of PROMOTE strategies. Posters, email pushes, micro-videos, and huddles are coordinated to focus on the same topic.

It is important to evaluate subcomponents of an intervention where possible before piloting. As there are several educational components to PROMOTE, these need to be individually evaluated. The purpose of this study was to (a) complete a pre-/post-test evaluation of the 7 min educational PROMOTE video to determine changes in staff knowledge, confidence, and intention to use the strategies, and (b) have LTC leaders, who typically train direct care staff on nutrition and hydration, review all PROMOTE educational components for their applicability to LTC. These data together will be used to not only demonstrate the value of individual PROMOTE educational components, but also to determine if revisions are required before launching the hydration program.

## 2. Materials and Methods

### 2.1. Participants

Participants viewing the educational video and completing the pre- and post-test based on this educational material were recruited primarily through the networks of the research team. In 2023, researchers sent an invitation email to their networks in LTC inviting home providers to share the link to the video and the pre- and post-tests with their staff. The information letter, consent, pre- and post-test questions, and video were embedded into the Qualtrics Insight Platform licensed to the University of Waterloo. Participants had the option on a separate Qualtrics form to provide their email address for a chance to win one of four CAD 50 draws for their completion of the questionnaires. This platform was open for 6 months. One researcher (RA) scanned all submissions to ensure they were legitimate (e.g., time between pre and post-test was sufficient to view the video, email addresses appeared legitimate, no repeat email addresses).

LTC leaders, who were responsible for direct-care staff education on nutrition and hydration, were recruited from the researchers’ network to review all the PROMOTE components. These key informants were sent an information letter and invited to review all the PROMOTE educational materials, providing feedback in the form of a short survey on each component, as well as an interview with a researcher (RA). They were each provided a gift certificate of CAD 50 to compensate them for their time. Ethics review was completed at the University of Waterloo (REB# 45334). Informed consent was obtained from all participants.

### 2.2. Procedure

**Figure 1:**
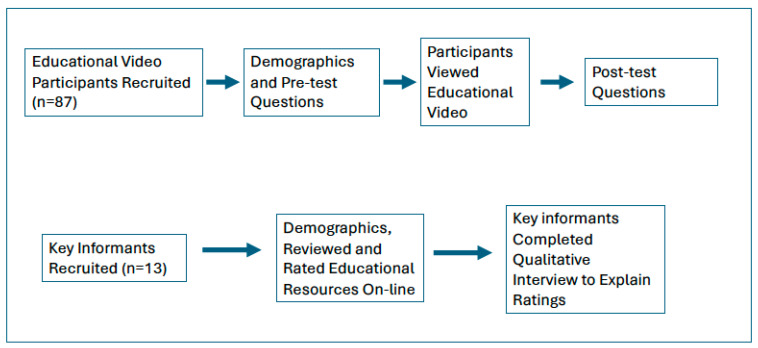
Overview of data collection procedures.

Figure 1 provides an overview of the flow of data collection. The 7 min PROMOTE staff training video had key messages of resident fluid requirements, reasons why residents may not drink enough, and the seven PROMOTE strategies to improve fluid intake between meals. Pre- and post-test questions were similar to those previously used in the “Wet your Whistle with Water” evaluation [18] and were created by the research team to assess staff perceptions, knowledge, confidence, and intention to use the strategies. Two pre-test-only questions determined participant perceptions on (a) whether staff in their LTC home routinely offered fluid between meals, and (b) whether in the past week they regularly offered fluids to residents between meals. Response options were strongly agree, agree, disagree, and strongly disagree. Five true/false questions were used pre- and post-test to determine staff change in knowledge with viewing the educational video. A question (scale rating 1–10) self-rating their level of knowledge on how to support residents with drinking was also asked. Staff perception on it being easy (rated 1) or hard (rated 10) to provide fluid between meals to residents was also determined. Finally, a question regarding staff confidence in helping residents who need assistance to drink and providing this assistance when performing other tasks with them was also asked pre- and post-test with response options of strongly agree, agree, disagree and strongly disagree. Two additional post-test questions were asked of participants, both using a rating scale of 1 (likely/very easy) to 10 (unlikely/very hard): (a) likelihood or intention to use PROMOTE strategies to improve resident hydration and (b) likelihood or intention to include PROMOTE strategies to increase fluid intake between meals as part of their work flow. Demographic questions were provided before the pre-test and included type of senior care (e.g., LTC, retirement home), age group, gender, fulltime work status, work role, experience, and geographic region in which they worked.

After providing consent, key informants were sent the educational materials and links to videos for their review. They were directed to a Qualtrics form where they could review the information letter again and provided consent before completing the demographic questions and rating the educational material. Demographics included the type of senior care where they were employed (e.g., LTC, retirement home), age group, gender, full-time work status, work role, years of experience in this sector, and region where they worked. They then rated each educational component on (a) usefulness (very, somewhat, not very, not at all useful), (b) feasibility of using the educational component in their work setting (very, somewhat, not very, not at all feasible), and (c) likelihood of using the educational component (yes, maybe, would not use, prefer not to say). Finally, participants were asked to provide a rating from 1 (low) to 10 (high) on the priority of their organization to implement the PROMOTE program. After completion of this rating online, researchers contacted the key informants for an interview to clarify their ratings. Verbal consent was provided at the time of the interview. These individual interviews were conducted virtually and positive feedback, challenges identified, and recommended changes for each educational component were elicited. Audio recordings of the virtual interview were used for analysis.

### 2.3. Data Analysis

Quantitative data were summarized as frequency count, mean, median and standard error of the mean (SEM). To determine change in knowledge and confidence of staff who reviewed the educational video, McNemar’s test and a paired *t*-test were used. Audio recordings from key informants were reviewed several times by two researchers to confirm content. Key informant interview data were summarized via content analysis [20] and tabulated by educational component. Data from both staff change in knowledge and confidence based on the educational video were compared qualitatively with key informant views on this educational resource. Statistical significance was determined at *p* < 0.05.

## 3. Results

### 3.1. Educational Video Participants

Seventy-six of the eighty-seven participants who reviewed the video and completed the pre- and post-test questions worked in LTC (87%). Participants were distributed across the age groups, with the highest number of participants being between 30 and 39 and between 50 and 64 years of age (*n* = 26 and 27, respectively). Almost all declared their gender to be woman (92% *n* = 80). Sixty-nine percent (*n* = 60) worked fulltime, and diverse roles were represented, with dietitian (32% *n* = 28) and healthcare aide (20% *n* = 17) being the most frequent. Only 8% (*n* = 7) had been working for less than 1 year, with the other work experience categories relatively evenly distributed. The sample was drawn from both Canada and the United States (Table 1).

A little more than half of staff participants (56%) who reviewed the educational video indicated thar they agreed with the statement ‘Most staff I know offer fluid to residents between meals’, while 12% strongly agreed, but 29% disagreed with this statement. Seven participants provided ‘prefer not to answer’ for the second pre-test question on current practice. Almost half (42.5%) who answered this question agreed that ‘In the past week, I regularly offered fluids to residents between meals’, but 26% disagreed and 14% strongly disagreed with this statement.

Table 2 summarizes educational video participant responses on the knowledge and confidence questions. The proportions provided in this table are those who answered true/false questions correctly or responded agree/strongly agree to statements at the pre-test and post-test. For the final two items in this table, the median is provided for the rating scales 1–10 used for these questions at pre- and post-test. There was a significant increase in the proportion indicating the correct response for two of the five knowledge questions. Two of the three remaining knowledge questions had high proportions with the correct answer at pre-test (“Food does not contribute to resident hydration” and “One way to increase fluid intake in residents is to offer fluid between meals”), while the question on thirst sensation had an increased proportion at post-test providing the correct response, although this difference was not statistically significant (*p* = 0.39). Participants were also statistically more likely to agree with the statement at post-test on being confident to support residents who needed help to drink in conjunction with other routines. There was a statistically significant difference between pre- and post-test rating (1 = low, 10 = high) for the statement ‘How would you rate your knowledge on how to support residents who do not drink enough?’ from a median of 8.00 (SEM 0.18) at pre-test to a median of 9.00 (SEM 0.13) at post-test. There was no change in the rating of the pre- and post-test (median 5.00 pre-test SEM 0.25, post-test SEM 0.30) for the statement ‘Providing residents with fluid between meals is (1 = easy to 10 = hard)’. Two further questions at post-test focused on intention to use PROMOTE. On a scale of 1 (likely) to 10 (unlikely), the median was 2.00 (SEM 0.27) for the statement ‘I intend to use PROMOTE strategies to increase resident fluid intake between meals’. Similarly high ratings (median 3.0 SEM 0.23) were found for the statement ‘Including PROMOTE strategies to increase resident fluid intake between meals will be (1 = very easy, 10 = very hard)’.

### 3.2. Key Informants

Of the 13 key informants who reviewed all educational materials, 69% worked in LTC, all were women, and 85% were dietitians. Almost half (47%) had worked for more than 16 years in this healthcare sector (Table 1). Key informants provided a median rating of 9 (SEM 0.42) on the question ‘How high a priority would it be for your organization to implement the PROMOTE program’ using a scale of 1 (low) to 10 (high). Table 3 outlines the perspectives of the key informants on the usefulness, feasibility, and likelihood of using each PROMOTE educational component. Both the staff educational and micro-videos were seen to be useful and feasible; 69% and 54% rated the educational video and micro-videos, respectively, as very useful. While 100% indicated that they would use the full-length video in the future, only 46% indicated the same for the micro-videos. Posters were seen to be feasible (very feasible, 54%; somewhat, 31%) and 85% responded that they would use the posters in the future. However, posters were more likely to be rated as only somewhat useful (general posters, 54%, and staff posters, 77%) or not useful (general posters, 23%; staff posters, 8%). Huddle discussions were seen to be potentially useful, but less feasible; 46% and 54% of participants rated them as very and somewhat useful, respectively, while only 31% indicated they were very feasible and 61% thought they were somewhat feasible. However, 77% of these key informants indicated that they would use the huddle discussions in training their home staff. Although 54% saw the email pushes as very feasible, and 46% said they were likely to use this educational component, key informants did not rate the usefulness of the email pushes very highly; 31% indicated that email pushes were very useful and 46% indicated that they were somewhat useful.

Key informant interviews provided insights into the challenges with the PROMOTE educational materials and recommendations for change. The comments provided in Table 4 help to interpret the feasibility and usefulness of educational components as summarized in Table 3.

Key informants could see using the educational video in team orientation or annual training and thought it provided a good overview of hydration. However, they thought this 7 min video was somewhat long and should be split into two short videos. Additional content around including fluid restrictions as an important consideration for staff to be aware of was recommended. Huddle discussions were seen to be interactive and an opportunity for staff to individualize strategies based on the checklist for reasons for why residents did not drink. However, they also noted this checklist would not be feasible in a single huddle and time to conduct routine huddles was a significant barrier. Reviewing huddle topics to ensure they were as concise as possible was recommended. Posters were seen as eye-catching and practical. Participants noted the time required to change posters on a weekly basis when located in several locations in the home. Some posters were also too dense with information. Recommendations for posters included using motivating messages and statistics, more graphics and colour, and providing general hydration posters after the 8-week PROMOTE campaign was completed to keep hydration as a topic of interest to staff and residents. Participants appreciated the shortness of the micro-videos and could see using them in huddles. Sending these short videos to staff via email was seen as less feasible. Some content changes were suggested for the micro-videos. Key informants liked that the email pushes were short and could be texted to staff, but barriers to their use were evident including a lack of email access, being ignored, or being considered an invasion of privacy. However, they could see using these messages to prep staff for upcoming huddles and considered how they could be provided to staff via organizational posting or inclusion in human resource email messages.

## 4. Discussion

### 4.1. Educational Video

Findings were supportive for the use of the 7 min educational video to improve the knowledge and confidence of staff to improve the fluid intake of residents living in retirement or long-term care homes. All the key informants indicated they would use this video in training staff on the hydration of residents. Participants who viewed the video had a significant change in two of five knowledge items (*p* < 0.05) between pre- and post-test. Their responses on a third question, although not statistically significant, showed an increase at post-test (thirst sensation question). The other two knowledge questions had a high correct response rate (>80%) at pre-test, suggesting that more challenging questions should be used in future evaluations. Confidence to support residents who required physical assistance to drink during routine care also significantly increased post-test. These same participants, however, indicated that they only agreed, or even disagreed (e.g., *n* = 29%), with statements at pre-test on current practice of offering fluid between meals to residents in the past week. In contrast, post viewing the video, these participants provided a high rating for intention to use PROMOTE strategies in the future. Coupled with the lack of change pre- and post-test on the question about difficulty providing fluid to residents between meals, those who viewed the 7 min video provided a slightly lower rating on ease of putting these strategies into place. These findings suggest that other factors beyond knowledge, confidence, and intention play an important role in the practice of providing fluid between meals to residents. For example, Cook et al. [15] identified that a home hydration policy is important to ensure the environmental and managerial support is provided to carry out strategies that promote resident hydration. Future evaluation of PROMOTE should include perception of managerial support.

Almost 70% of key informants indicated the 7 min staff educational video was useful. To improve the feasibility (54% rated very feasible) of the video, they recommended segmenting the content into two shorter videos that could be used in team meetings and other routine opportunities for staff education, as key informants indicated that some staff may not have access to a personal device to view the video. Of note, staff education that has been shown to improve hydration knowledge in prior research included longer sessions than the current study [16,17]. Despite training with I-Hydrate being well received and improving staff knowledge, practice change was not observed [16]. The I-Hydrate authors suggested that lack of training for all staff was part of the issue and recommended the use of short huddle meetings and posters to reinforce key concepts [16]. E-learning and videos have the flexibility of being available when the user would like to view the material [17], and when videos are short, they may be more likely to be viewed by more staff [18]. Feasibility can be further enhanced by providing computer stations or tablets for staff to use during their time on shift, or where available, to play the video on screens in public areas of the homes. Viewing of videos can also be encouraged by making training mandatory [17]. An efficient and effective training module, such as the short PROMOTE staff video, could encourage viewing by more staff [16,18].

### 4.2. Other Educational Components

Key informants provided insights into the usefulness and feasibility of the other PROMOTE educational components. The short micro-videos were seen to be both useful and feasible and could be used at team meetings to reinforce key messages as they were approximately two minutes in length. However, the likelihood of using the micro-videos in the future was lower than for the 7 min educational video, potentially as determining how to implement several very short videos would require different processes than current in-service training for staff. Huddle topics and discussions for team meetings were viewed as useful and key informants were interested in using these in the future, but feasibility was a concern. Key informants also recommended discussing fluid restrictions in educational materials.

One of the huddle topics was a checklist for causes of poor fluid intake in individual residents—although seen as useful, there was concern about the feasibility of staff completing this checklist for all residents in a single huddle meeting. Other hydration toolkits include individual resident assessment on risks for poor fluid intake to target interventions [15,19], and thus this appears to be a worthwhile topic to be included in huddles. Modifying this activity to only focus on those residents who currently do not drink enough may make it more manageable for LTC homes, as well as breaking up the completion of the checklist over more than one huddle or team meeting. This, however, would necessitate staff having a good awareness of individual resident fluid intake by routinely monitoring and tracking consumption. Think Drink [15] and the Hydration Toolkit [19] use team meetings to raise awareness and reinforce concepts, while using a booklet to provide a range of interventions to promote hydration. It is not clear what percentage of staff participated in these sessions or used these information booklets. I-Hydrate used in-person training sessions which were offered several times throughout the year; however, participation varied in these in-person sessions based on staff availability [16]. The PROMOTE concept of multiple huddle meeting discussions and micro-videos on the same topics takes the place of an educational booklet and longer workshops and provides training for staff on an on-going basis. This would require a hydration champion in the LTC home to implement PROMOTE. This could be a full-time staff member who is respected and considered an influential team member. They could be trained on how to roll out the PROMOTE intervention, as well as behaviour change techniques. Cole [19] acknowledges the need for a hydration champion to keep staff focused on using hydration strategies in practice over time.

Consistent with prior work, posters were seen by key informants to be feasible, but not as useful for motivating change as other educational components [9]. Hydration posters have been used to raise awareness in homes [16,19], but their effectiveness is unknown as compared to other components of an intervention. Key informants suggested ways to expand on the current PROMOTE posters to make them more appealing and motivating and continue to keep hydration a topic of interest after an educational campaign. Posters and micro-videos on screens in common areas would also be a way to reach residents and family members. The implementation of PROMOTE should also consider how to involve residents and family members to support resident hydration. The key informants’ recommendations for the various PROMOTE components can be readily incorporated into the next version of the program. Consistent with prior work [19] and based on the key informants’ variability in intention to use the different PROMOTE educational materials found in this study, homes using PROMOTE should be encouraged to pick and choose what materials work best for their home and staff.

### 4.3. Limitations

A high proportion of participants who reviewed and completed the evaluation of the educational video were dietitians, which likely influenced results, as some knowledge items already had a high positive response rate at pre-test. There were also some ‘prefer not to answer’ responses, potentially as these participants were not involved in direct care around offering fluids to residents. Key informants provided good insights into the educational components of PROMOTE, but this is not equivalent to having direct-care staff themselves identifying what the most motivational or impactful components of the PROMOTE educational materials to support their improved practice are. A future effectiveness study on PROMOTE should include evaluation from direct-care staff on these individual educational components.

## 5. Conclusions

Staff education is essential for implementing lasting behaviour changes in LTC, such as in supporting resident fluid intake to prevent dehydration. A 7 min educational video, tailored to staff, improved knowledge and confidence to support residents who need physical help to drink. Key informants confirmed the usability of this staff video, as well as other educational components of the PROMOTE intervention, and provided recommendations to improve these resources. Educational video reviewers and key informant participants also indicated high intention to use the PROMOTE evidence-based strategies to improve resident hydration. Despite these positive outcomes, participants also noted that systemic challenges can impact fluid offerings between meals and thus resident hydration. Future work evaluating PROMOTE should include the evaluation of all educational components with direct care staff and residents, considering the effects on outcomes including staff behaviour change in practice and resident hydration.

## Figures and Tables

**Table 1:** Participant demographics.

	Educational Video Participants *n* = 87	Key Informant Participants *n* = 13
Demographic Categories	n (%)	n (%)
*Area of Employment*		
Long-term care or nursing home	76 (87)	9 (69)
Assisted living	4 (5)	--
Retirement home/ independent living	3 (3)	--
Other form of older adult care	4 (5)	4 (31)
*Age Group (years)*		
20–29	12 (14)	3 (23)
30–39	26 (30)	3 (23)
40–49	19 (22)	3 (23)
50–64	27 (31)	4 (31)
≥65 years of age	3 (3)	--
*Gender*		
Woman	80 (92)	13 (100)
Man	6 (7)	--
Transgender Woman	1 (1)	--
*Work*		
Full-time	60 (69)	8 (62)
Part-time/casual	27 (31)	5 (38)
*Work Roles*		
Dietitian	28 (32)	11 (85)
Healthcare aide/ personal support worker	17 (20)	--
Management	12 (14)	--
Food service	3 (3)	--
Nursing	13 (15)	--
Other	14 (16)	2 (15)
*Seniors’ Living Work Experience*		
<1 year	7 (8)	--
1–7 years	34 (39)	5 (38)
8–15 years	17 (20)	2 (15)
16+	29 (33)	6 (47)
*Geographic Location*		
Western Canada	41 (47)	7 (54)
Eastern Canada	25 (29)	4 (31)
United States	21 (24)	2 (15)

**Table 2:** Participants responding correctly to survey questions on educational video (*n* = 87).

Question	Pre-Test	Post-Test
**Residents who require thickened beverages are at high risk for dehydration as compared to those who drink thin fluids.**	**68% ^a^**	**82% ^a^**
Food does not contribute to resident hydration needs.	80% ^b^	83% ^b^
**Residents will drink more when they are with their family or friends.**	**72% ^a^**	**84%**
One way to increase fluid intake in residents is offering fluids between meals.	86%^a^	86%
The thirst sensation is increased in older adults.	71% ^b^	79%
**For residents needing assistance to drink, I am confident that I can help them to drink when I am doing other things with them.**	**68% ^c^**	**76%**
**With 1 being low and 10 being high…How would you rate your knowledge on how to support residents who do not drink enough?**	**8.00 ^d^**	**9.00**
Where 1 is easy to do and 10 is hard to do…Providing residents with fluid between meals is…	5.00 ^d^	5.00

Note. Emboldened questions are significant at *p* < 0.05. ^a^ Percentage of participants responding to correctly true for a true/false question. ^b^ Percentage of participants responding to correctly false for a true/false question. ^c^ Percentage of participants agreeing or strongly agreeing with the statement. ^d^ Median based on a scale rating of 1–10.

**Table 3:** Key informants’ rating of the usefulness of, feasibility of, and intent to use PROMOTE educational components (*n* = 13).

Ratings	Educational Components (%)					
	Educational Video	Huddles	General Posters	Staff Posters	Micro-Videos	Email Pushes
**Usefulness**						
*Very*	69% ^a^	46%	23%	15%	54%	31%
*Somewhat*	31%	54%	54%	77%	38%	46%
*Not very*	--	--	23%	8%	8%	23%
			**Posters**			
**Feasibility**						
*Very*	54%	31%	54%		61%	54%
*Somewhat*	46%	61%	31%		8%	23%
*Not very*	--	8%	15%		23%	15%
*Not at all*	--	--	--		8%	8%
**Intention to use**						
*Yes*	100%	77%	85%		46%	46%
*Maybe*	--	15%	15%		31%	23%
*No*	--	8%	--		15%	31%
*Prefer not to say*	--	--	--		1%	

^a^ Table values are the proportion of participants.

**Table 4:** Key informant perspectives and recommendations for educational material.

Type	Positive Comments	Challenges Identified	Recommendations
Educational Video	Good overview Use at team meetings, between shifts Use in general orientation, annual training	7 min too long Omits information on fluid restriction Feasibility affected by staff devices Feasibility affected by paid time to complete	Split into 2 videos Include further content (e.g., fluid restriction) Add statistics to motivate Make more interactive
Huddle Discussions	Promote staff collaboration Good range of topics Interactive, fun elements and get staff thinking Opportunity to individualize strategies to residents with checklist	Checklist on type of challenges for residents may not be feasible Time to conduct huddles Puts onus on individuals to reach all staff	Make concise
Posters	Concrete ideas Practical Eye-catching Good variety Reinforce learning Could be used in a variety of places in the home	Staff posters have too much information Requires work to change weekly if in multiple sites	Change colour for each topic Add graphics, borders, statistics Need new posters for main messages to be used after the campaign Create a poster for use in resident room Include motivating messages
Micro-videos	Short, flexible for use Useful for team meetings, huddles, shift change Good flow of topics	May not be useful for all staff; do not have email Feasibility based on device availability, time to watch videos during shift	Improve terminology Add details on thickened beverages Provide detail on frequency of offers
Email pushes	Short reminder Can text vs. email prepare for huddle discussions	Staff may ignore Lack of email access Redundant messages Not experiential Text may be considered an invasion of privacy	Post on organizational websites Use for leadership, add to a human resource email message Include prompts to help staff empathize with residents Offer a text version

## Data Availability

The datasets presented in this article are not readily available because consent for sharing beyond the researchers involved in this study was not requested of participants. Summarized data are available from H Keller.
